# Human-Delivered Conversation Versus AI Chatbot Conversation in Increasing Heart Attack Knowledge in Women in the United States: Quasi-Experimental Studies

**DOI:** 10.2196/73184

**Published:** 2025-10-17

**Authors:** Diane Dagyong Kim, Jingwen Zhang, Kenji Sagae, Holli A Devon, Thomas J Hoffmann, Lauren Rountree, Yoshimi Fukuoka

**Affiliations:** 1 Department of Communication, University of California, Davis, 177 Kerr Hall, Davis, CA, 95616, United States, 1 530-754-1472; 2 Department of Communication, Department of Public Health Sciences, University of California, Davis, Davis, CA, United States; 3 Department of Linguistics, University of California, Davis, Davis, CA, United States; 4 UCLA School of Nursing, University of California, Los Angeles, Los Angeles, CA, United States; 5 Department of Epidemiology & Biostatistics, University of California, San Francisco, San Francisco, CA, United States; 6 Massachusetts General Hospital, Boston, MA, United States; 7 Department of Physiological Nursing, University of California, San Francisco, San Francisco, CA, United States

**Keywords:** artificial intelligence, natural language processing, conversational agent, large language model, chatbot, health education, heart disease, heart attack, knowledge, women

## Abstract

**Background:**

Artificial intelligence (AI) chatbots, driven by advances in natural language processing, can analyze and generate human language through computational linguistics and machine learning. Despite the rapid development of large language models, little investigation has been conducted to assess whether AI chatbot-delivered educational conversations can achieve a similar level of efficacy as human-delivered conversations.

**Objective:**

This study aims to evaluate and explore the potential efficacy of human-delivered conversations versus AI chatbot conversations in increasing women’s knowledge and awareness of symptoms and response to a heart attack in the United States.

**Methods:**

This is a secondary analysis of 2 datasets collected from the AI Chatbot Development Project. Women aged 25 years or older were recruited through flyers and social media. The first dataset contained conversational data where a research interventionist engaged in educational conversations with participants (human dataset), whereas the second dataset contained conversational data where an AI chatbot named HeartBot engaged in the same educational conversations with participants (HeartBot dataset). Knowledge and awareness of symptoms and response to a heart attack were measured at the pre- and post-interaction with either the human or HeartBot. Perceived message effectiveness and conversational quality were measured at the post-survey. Ordinal logistic regression analyses were conducted to explore factors predicting participants’ knowledge, adjusting for age, race or ethnicity, intervention group type, education, word count, message effectiveness, and message humanness.

**Results:**

A total of 171 participants (mean age=41.06 y, SD=12.08) in the Human dataset and 92 participants (mean age=45.85 y, SD=11.94) in the HeartBot dataset completed the study. Both human-delivered conversations and HeartBot conversations were associated with significant improvements in participants’ ability to recognize heart attack symptoms (adjusted odds ratio [AOR] 15.19, 95% CI 8.46‐27.25, *P*<.001; AOR 7.18, 95% CI 3.59‐14.36, *P*<.001), differentiate between symptoms (AOR 9.44, 95% CI 5.60‐15.91, *P*<.001; AOR 5.44, 95% CI 2.76‐10.74, *P*<.001), call emergency services (AOR 6.87, 95% CI 4.09‐11.55, *P*<.001; AOR 5.74, 95% CI 2.84‐11.60, *P*<.001), and seek emergency care within 60 minutes of symptom onset (AOR 8.68, 95% CI 4.98‐15.15, *P*<.001; AOR 2.86, 95% CI 1.55‐5.28, *P*<.001), even after adjusting for covariates. Comparing the 2 datasets via interaction tests showed a statistically significant improvement in human-delivered conversations versus HeartBot conversation for all but the calling an ambulance question (*P*=.09).

**Conclusions:**

The study’s findings provide new insights into the fully automated AI HeartBot, compared to the human-driven text message conversations, and suggest that it has potential in improving women’s knowledge and awareness of heart attack symptoms and appropriate response behaviors. Nevertheless, the current evidence remains preliminary. A randomized controlled trial is warranted to validate this study’s findings.

## Introduction

Heart disease remains the leading cause of death for women in the United States [1]. Over 60 million women in the United States are living with heart disease [2]. Despite public campaigns, such as “Go Red for Women” by the American Heart Association [3], awareness of heart disease as the leading cause of death among women has declined from 65% in 2009 to 44% in 2019 [4]. The most significant declines have been observed among Hispanic women, Black women, and younger women [5]. Given this troubling trend, there is an urgent need for alternative and scalable approaches to increase knowledge and awareness of heart disease in women.

An artificial intelligence (AI) chatbot could be one of the promising approaches to improve women’s awareness of heart disease. AI chatbots are built on natural language processing, natural language understanding, and machine learning. Several systematic reviews have investigated the usability and potential efficacy of AI chatbots in managing patients with various health conditions. Overall, AI chatbot–based interventions have shown the potential to improve mental health, such as depressive and anxiety symptoms; promote healthy diets; and enhance cancer screenings [6-13]. However, the fast-growing capabilities of AI chatbots raise questions about their ability to compete with human cognitive and emotional intelligence. Yet, only a few randomized controlled trials (RCTs) have directly compared the efficacy of AI chatbots to that of human agents. For example, the studies reported that the AI chatbot offers efficacious counseling to patients with breast cancer comparable to that of health professionals [14-16]. To the best of our knowledge, no clinical trial exists on whether an AI chatbot is effective in increasing women’s heart attack awareness and knowledge. Further empirical investigation is needed to more comprehensively evaluate the efficacy of AI chatbots compared to human agents.

Our research team initiated an AI chatbot development project aimed at increasing women’s knowledge and awareness of heart attack. As a first step, we collected a conversational dataset in which a research interventionist texted each participant with educational content on heart health (Human dataset) over 2 days. We subsequently developed and tested a fully automated SMS text messaging–based AI chatbot system named HeartBot, available 24/7, designed to achieve similar objectives and collected a conversational dataset between HeartBot and participants. The detailed study design, including HeartBot’s development mechanism and algorithmic structure, is published elsewhere [17]. This project presents a valuable opportunity for a comparative secondary analysis, and this paper focuses specifically on examining the outcomes of the 2 studies.

The aim of this secondary data analysis is to evaluate and explore the potential efficacy of the 2 heart attack education interventions (SMS text messaging intervention delivered by a human research interventionist vs an AI chatbot [hereafter HeartBot]) in community-dwelling women without a history of heart disease. The primary outcome is participants’ knowledge and awareness of symptoms and response to a heart attack. In addition, we examined differences in participants’ evaluations of user experience and conversational quality across the 2 formats by assessing message effectiveness, message humanness, naturalness, coherence, and conversational metrics. Our study is among the first to provide a detailed understanding and multidimensional comparison of human-delivered and automated AI chatbot interventions in the context of heart attack education. These findings contribute new insights into the relative strengths of human- and AI-driven health communication, offering practical guidance for designing more effective education and behavior change programs.

## Methods

### Study Design and Sample

This was a secondary analysis on 2 datasets collected from the AI Chatbot Development Project conducted from September 2022 to January 2024 [17]. The aims of the AI Chatbot Development Project are to conduct a series of studies to develop a fully automated AI chatbot to increase knowledge and awareness of heart attack in women in the United States. After convening a multidisciplinary team, we developed a knowledge bank using the clinical guidelines, published papers, and American Heart Association’s “Go Red for Women” materials [3] to develop the content of the conversation. Then, we conducted a Wizard of Oz experiment with the Human dataset cohort, where participants interacted with a system they believed to be autonomous but was operated by a research interventionist [18], to test the content and aid in the development of a text-based HeartBot with natural language capabilities. The research interventionist, who was a master-prepared, experienced cardiovascular nurse, served as the research interventionist to interact with the participants through SMS text messaging (phase 1: Human dataset).

After the first study (phase 1), we developed a fully automated AI chatbot, the HeartBot, to deliver the intervention through SMS text messaging (phase 2: HeartBot dataset). The detailed design of the project, including the protocol, participant eligibility criteria, and description of the HeartBot platform, was published elsewhere [17].

The eligibility criteria for both studies were women (1) being aged 25 years or older, (2) living in the United States, (3) having access to the internet to complete the online survey and a cell phone with SMS text messaging capabilities, (4) having no history of cognitive impairment or history of heart disease or stroke, and (5) who were not health care professionals or students. The eligibility criteria were consistent throughout the 2 studies. Participants in both studies were mainly recruited from Facebook (Meta) and Instagram (Meta) from September 2022 to January 2023 and from October 2023 to January 2024, respectively.

### Procedure and Interventions

For the Human dataset (phase 1), participants who were interested in the study were recruited online and underwent screening to confirm eligibility. Eligible participants provided written informed consent prior to enrollment and completed a baseline survey online. Then, participants engaged in 2 online conversation sessions over the course of 2 days over a week with a research interventionist, with each session covering educational content related to heart attack symptoms and response. Table 1 presents the content of heart attack topics used in both studies. After having a text conversation, participants completed a post online survey to measure knowledge and awareness of symptoms and response to a heart attack, message effectiveness, message humanness, conversation naturalness and coherence, and perception of chatbot identity. Participants were provided with a $40 Amazon e-gift card upon completion of all study procedures.

**Table 1.** Content of heart attack topics in the artificial intelligence (AI) Chatbot Development Project.

	Phase 1: Human dataset	Phase 2: HeartBot dataset
Session 1	Greetings What is heart attack Symptoms of heart attacks Leading cause of death for women in the United States Gender factors of heart attacks How angina happens Risk factors for heart disease Female-specific risk factors for heart disease Racial risk factors of heart disease	Greetings Participants’ name retrieval Knowledge on heart attacks Symptoms of heart attacks Leading cause of death for women in the United States Gender factors of heart attacks First action Importance of calling 911 Waiting duration Treatment of heart attacks Action during waiting for 911 Risk factors for heart disease Female-specific risk factors for heart disease Racial risk factor for heart disease Multiple-choice quiz questions Further questions to ask End of the conversation
Session 2	First action Importance of calling 911 Waiting duration Tests to diagnose a heart attack Medicines for heart attack Operational procedures for treating heart attack Prevention of heart attacks End of the conversation	Not applicable

We conducted a follow-up phase, developing and evaluating the text-based AI chatbot called HeartBot. A comprehensive description of the HeartBot was published previously [17]. In short, HeartBot was designed as a rule-guided, SMS-based conversational agent that delivers pre-authored educational messages in a structured format. We implemented it on the Google Dialoflow CX platform and linked it to Twilio [19] for text messaging conversation based on the intents and entities paradigm [20]. HeartBot identified the general intent of each incoming message and responded with an appropriate, scripted reply. Although HeartBot can recognize a range of user inputs, its responses are intentionally constrained to maintain accuracy and consistency in delivering heart disease education. HeartBot engaged in 1 conversation session with the participants. We decided to condense the conversational messaging to only 1 session to reduce the chance of participant attrition and make sure participants can receive all educational information within 1 interaction. In contrast to the first phase of the project (Human dataset), 3 topics (how angina happens, medicines for heart attack, and operational procedures for treating heart attack) were dropped in the second phase (HeartBot dataset), and 2 quiz questions were included at the end of the conversation to assess participants’ retention of key knowledge outcomes. Participants then completed the post online survey and received a $20 Amazon e-gift card. Both studies used the same questionnaires for both the baseline online survey and the post online survey to measure knowledge and awareness of symptoms and response to a heart attack, hosted on a secure online tool called Research Electronic Data Capture [21].

### Ethical Considerations

The first and second studies (phases 1 and 2) were conducted in accordance with the ethical standards outlined in the Declaration of Helsinki. Institutional Review Board approvals were obtained from the University of California, Los Angeles (approval number: 23-000878), for the first study and from University of California, San Francisco (approval number: 23-29793), for the second study. For both studies, all participants provided written informed consent prior to study enrollment. Participation was voluntary, and participants were informed that they could withdraw at any time without penalty. All collected data were deidentified prior to analysis, and no personally identifiable information was retained. Data were stored on secure, password-protected servers accessible only to the research team. As part of the compensation, participants in the first and second studies who completed all study requirements received a $40 e-gift and a $20 e-gift card, respectively.

### Measures

#### Primary Outcomes: Knowledge and Awareness of Symptoms and Response to Heart Attack

To assess the potential efficacy of a conversational intervention to increase the knowledge and awareness of symptoms and response to a heart attack, we adapted a previously validated scale [22,23]. These items have also been used in prior research involving women from diverse backgrounds to ensure broad applicability [24-26]. Participants were asked the following 4 questions on a scale of 1-4 where 1 indicated “not sure” and 4 indicated “sure”: (1) “How sure are you that you could recognize the signs and symptoms of a heart attack in yourself?,” (2) “How sure are you that you could tell the difference between the signs or symptoms of a heart attack and other medical problems?,” (3) “How sure are you that you could call an ambulance or dial 911 if you thought you were having a heart attack?,” and (4) “How sure are you that you could get to an emergency room within 60 minutes after onset of your symptoms of a heart attack?*”* The same questions were asked before and after the interaction with the research interventionist and HeartBot. A higher score indicates better knowledge and awareness of symptoms and response to a heart attack.

#### Other Measures 

##### Overview

We used the AI Chatbot Behavior Change Model [27] to assess user experience and conversational quality as key dimensions of effective chatbot communication. Message effectiveness and perceived message humanness were assessed to capture how participants interpreted and responded to the HearBot’s messages. These key measures were selected to better understand how participants evaluated the interaction and how specific communication features may have influenced their experience.

##### User Experience: Message Effectiveness

Based on the AI chatbot Behavior Change Model [27], message effectiveness is conceptualized as an aspect of the broader category of “user experiences,” which measures the level of usefulness and convenience in chatbot conversations. Participants completed a post-survey measure known as the *Effectiveness Scale*, a semantic-differential scale originally developed based on prior research [28,29]. The scale consists of 5 items, including bipolar adjective pairs (effective vs ineffective, helpful vs unhelpful, beneficial vs not beneficial, adequate vs not adequate, and supportive vs not supportive). Each item was rated on a 7-point Likert scale, 1 being the negative pole (eg, “ineffective”) and 7 being the positive pole (eg, “effective”). The scores for each item were summed and averaged to create a mean composite score, with higher scores indicating greater perceived effectiveness of the messages.

##### Conversational Quality

###### Message Humanness

The humanness of chatbot messages in the AI chatbot Behavior Change Model [27] is conceptualized as a part of “conversational quality,” a construct that reflects the perceived human-likeness and naturalness of chatbot interactions. To evaluate participants’ impressions of the messages sent by the research interventionist and HeartBot, participants completed the *Anthropomorphism Scale* [30] during the post-survey. The scale includes 5 pairs of bipolar adjectives (natural vs fake, humanlike vs machine-like, conscious vs unconscious, lifelike vs artificial, and adaptive vs rigid). Participants rated each pair on a 7-point Likert scale, where 1 indicated the first adjective in the pair (eg, “natural”) and 7 indicated the second adjective (eg, “fake”). The scores for each item were summed and averaged to create a mean composite score. The higher scores indicate a greater perception of chatbot messages as more mechanical or artificial.

###### Conversational Naturalness and Coherence

Conversational quality can be assessed by participants’ subjective evaluation of the conversation’s naturalness and coherence [27]. To evaluate conversational quality, participants were asked to answer the following question in the post-survey: “Overall, how would you rate the conversations with your texting partner?” The response options are as follows: (1) Very unnatural, (2) Unnatural, (3) Neutral, (4) Natural, and (5) Very natural. Participants were also asked to answer the following question in the post-survey: “Overall, how would you rate the messages you received?*”* The response options are as follows: (1) Very incoherent, (2) Incoherent, (3) Neutral, (4) Coherent, and (5) Very coherent.

###### Conversational Metrics

Objective content and linguistic analyses of conversations can be used to evaluate specific dimensions of conversations, such as the length of conversations and the amount of information exchanged [27]. To measure these dimensions, the Linguistic Inquiry and Word Count (LIWC-22; Pennebaker Conglomerates) software [31] was used to process and quantify the total word count of a conversation between the participant and the research interventionist or HeartBot. The number of words used by each agent (participant, research interventionist, and HeartBot) was separately measured to process individual contributions within each conversation.

###### Perception of Chatbot Identity (Human vs AI Chatbot)

At the end of the intervention, we asked the question: *“*Do you think you texted a human or an artificial intelligent chatbot during your conversation?*”* Participants were asked to select either of the 2 response options, which were dichotomous: (1) human or (2) artificial agent.

###### Sociodemographic, Past Chatbot Use, and Cardiovascular Risks

Self-reported sociodemographic information (ie, age, race or ethnicity, education, household income, marital status, and employment status) and cardiovascular risks (ie, smoking history, prescribed blood pressure, cholesterol, and diabetes medication intake, and family history of heart disease) were collected in the baseline survey online. The cardiovascular risk factor variables were selected based on the latest clinical guidelines [32]. In addition, the question “Have you used any chatbot in the past 30 days?*”* was used to assess past AI chatbot use experience. The participants were asked to select either Yes or No.

### Statistical Analysis

We conducted a descriptive analysis to calculate counts and percentages, or means and SD for sociodemographic characteristics, past chatbot use, and cardiovascular risks. To compare the 2 datasets, we performed independent *t* tests to assess mean differences for continuous variables and used *χ*^2^ tests to examine group distributions. We first conducted Wilcoxon signed-rank tests to evaluate for statistically significant changes in heart attack knowledge and awareness outcome responses (not sure, somewhat not sure, somewhat sure, and sure) between the baseline and the post-interaction, within the Human dataset (phase 1) and HeartBot dataset (phase 2). Then, to adjust for potential confounders, we fit a series of ordinal mixed-effects models using the R (version 4.1.0; The R Foundation for Statistical Computing) [33] package ordinal v2022.11.16 [34], for each of the 4 knowledge questions as outcomes. We first fit these models stratified by Human dataset (phase 1) and HeartBot dataset (phase 2), and adjusting for fixed effects of post (vs pre; the primary coefficient of interest for these models, indicating whether each of the 2 interventions was successful), White (vs non-White), age, interaction group type, education, number of words used by the participants, mean text message effectiveness and humanness of scores, and a random effect for individual. We then fit a model on the entire dataset additionally adjusting for HeartBot (vs Human), and the interaction between HeartBot and post timepoint (ie, whether HeartBot is more effective than human; the primary coefficient of interest for this model). As an attempted sensitivity analysis, we tried to fit a mixed effects multinomial logistic regression model in Stata (version 16.1; StataCorp LLC) [35] via the generalized structural equations command, but the models would not converge (likely owing to the small sample size and increased number of parameters to estimate compared to an ordinal logistic regression model). A 2-sided test was used with significance set at *P*<.05.

## Results

### Sample Characteristics

Multimedia Appendix 1 shows screening, enrollment, and follow-up of the study participants. A total of 171 participants in the Human dataset (phase 1) and 92 participants in the HeartBot dataset (phase 2) completed the study. Table 2 presents the baseline sample characteristics for the 2 datasets. The mean age (SD) of participants was 41.06 (12.08) years in phase 1 and 45.85 (11.94) years in phase 2. In the Human dataset (phase 1), participants were primarily Black/African American (n=70, 40.9%), college graduates (n=103, 60.3%), and earning moderate-to-high income (n=68, 39.8%). Participants in the HeartBot dataset (phase 2) were primarily White (n=37, 40.2%), college graduates (n=66, 71.7%), and earning moderate-to-high income (n=39, 42.4%). A majority of participants in the Human dataset (phase 1) reported having experience in using chatbot (n=96, 56.1%) as did participants in the HeartBot dataset (phase 2; n=53, 57.6%).

**Table 2.** Study sample characteristics: sociodemographic, previous chatbot use, cardiovascular risks.

Characteristic	Human dataset (n=171)	HeartBot dataset (n=92)	*P* value
Age (years), mean (SD)/[range]	41.06 (12.08)/[25.0‐76.0]	45.85 (11.94)/[26.0‐70.0]	.002
Race/ethnicity, n (%)			.097
Black/African American (non-Hispanic)	70 (40.9)	22 (23.9)
Hispanic/Latino	29 (17.0)	19 (25.0)
Asian	10 (5.8)	6 (6.5)
White (non-Hispanic)	50 (29.2)	37 (40.2)
American Indian/Native Hawaiian/more than 1 race/ethnicity	12 (7.0)	8 (8.7)
Education, n (%)			.06
Completed some college course work, but did not finish or less	68 (39.8)	26 (28.3)
Completed college/graduate school	103 (60.3)	66 (71.7)
Household income, n (%)			
<US $40,000/don’t know	59 (34.5)	23 (25.0)	.24
US $40,001-$75,000	44 (25.7)	30 (32.6)
>US $75,000	68 (39.8)	39 (42.4)
Marital status, n (%)			.63
Never married	46 (26.9)	21 (22.8)
Currently married/cohabitating	108 (63.2)	59 (64.1)
Divorced/widowed	17 (9.9)	12 (13.0)
Employment status, n (%)			.15
Full-time/part-time	108 (63.2)	56 (60.9)
Unemployed/homemaker/student	42 (24.5)	17 (18.5)
Retired/disabled/other	21 (12.3)	19 (20.7)
Chatbot use (eg, Amazon’s Alexa, Google Assistant, Siri, Facebook Messenger bot etc) in the past 30 days, n (%)			.82
Yes	96 (56.1)	53 (57.6)
No	75 (43.9)	39 (42.4)
Cardiovascular risks, n (%)			
Smoked at least one cigarette in the last 30 days			.08
Yes	14 (8.2)	14 (15.2)	
No	157 (91.8)	78 (84.8)	
Blood pressure medication			.045
Yes	71 (41.5)	25 (27.2)
No/don’t know	100 (58.5)	67 (72.8)
Cholesterol medication			.69
Yes	62 (36.3)	29 (31.5)
No/don’t know	109 (63.8)	63 (68.5)
Diabetes medication			.91
Yes	23 (13.5)	12 (13.0)
No/don’t know	148 (86.6)	80 (87.0)
Family history of heart disease/stroke			.11
Yes	38 (22.2)	13 (14.1)
No/don’t know	133 (77.8)	79 (85.9)

### Changes in Knowledge and Awareness of Heart Disease

Table 3 presents the results of Wilcoxon signed-rank tests examining pre- to post-changes in 4 knowledge and awareness of heart disease outcomes. Supplementary Tables S1-S3 (in Multimedia Appendix 2) present the full ordinal logistic regression models: Table S1 for the human-delivered conversations, Table S2 for HeartBot conversations, and Table S3 for the combined data. Overall, Wilcoxon signed-rank tests revealed a significant increase in knowledge and awareness of heart disease across all 4 outcome measures following interactions with both research interventionist and HeartBot (human-delivered conversations: all *P*<0.001; HeartBot conversations: *P*<.001 for Q1-Q3 and *P*=.002 for Q4).

**Table 3.** Change in participants’ knowledge and awareness of symptoms and response to heart attack between pre- and post-human conversation (n=171) and pre- and post-HeartBot conversation (n=92) for 4 outcome questions.

	Human-delivered conversations (n=171)			HeartBot conversations (n=92)		
	Pre-human conversation (%)	Post-human conversation (%)	*P* value^a^ (%)	Pre-HeartBot conversation (%)	Post-HeartBot conversation (%)	*P* value^a^
Q1: Recognizing signs and symptoms of a heart attack			<.001			<.001
1: Not sure	17.50	1.20		26.10	3.30	
2: Somewhat unsure	37.40	4.10		34.80	30.40	
3: Somewhat sure	36.30	56.10		35.90	43.50	
4: Sure	8.80	38.60		3.30	22.80	
Q2: Telling the difference between the signs or symptoms of a heart attack and other medical problems			<.001			<.001
1: Not sure	26.90	6.40		30.40	8.70	
2: Somewhat unsure	48.00	20.50		41.30	38.00	
3: Somewhat sure	19.30	50.90		26.10	43.50	
4: Sure	5.80	22.20		2.20	9.80	
Q3: Calling an ambulance or dialing 911 when experiencing a heart attack			<.001			<.001
1: Not sure	19.90	1.80		14.10	3.30	
2: Somewhat unsure	24.60	7.00		21.70	14.10	
3: Somewhat sure	22.80	23.40		34.80	21.70	
4: Sure	32.70	67.80		29.30	60.90	
Q4: Getting to an emergency room within 60 minutes after onset of symptoms of a heart attack			<.001			.002
1: Not sure	18.10	1.80		18.50	6.50	
2: Somewhat unsure	22.80	7.00		18.50	13.00	
3: Somewhat sure	26.30	18.70		31.50	33.70	
4: Sure	32.70	72.50		31.50	46.70	

a Wilcoxon matched pairs tests were conducted.

Table 4 shows the adjusted odds ratios (AORs) from a series of ordinal logistic regression analyses for predicting each knowledge question for the Human dataset (phase 1). In the Human dataset (phase 1), after controlling for age, ethnicity, education, message effectiveness, message humanness, and chatbot use history, the human-delivered conversations improved participants’ knowledge and awareness in recognizing the signs and symptoms of a heart attack response (AOR 15.19, 95% CI 8.46‐27.25, *P*<.001), telling the difference between the signs or symptoms of a heart attack response (AOR 9.44, 95% CI 5.60‐15.91, *P*<.001), calling an ambulance or dialing 911 during a heart attack response (AOR 6.87, 95% CI 4.09‐11.55, *P*<.001), and getting to an emergency room within 60 minutes after onset of symptoms response (AOR 8.68, 95% CI 4.98‐15.15, *P*<.001). In the HeartBot dataset (phase 2), these effects were generally reduced but still substantially improved (see Table 4; full model in Multimedia Appendix 2), for example, in recognizing the signs and symptoms questions (AOR 7.18, 95% CI 3.59-14.36, *P*<.001). A formal interaction test showed a statistically significant improvement of Human versus HeartBot dataset for all but the third question (calling an ambulance; *P*=.09) as shown in Table 4 (Table S3 in Multimedia Appendix 2). We could not adjust for word count, as all human-delivered conversations in the Human dataset (phase 1) were longer than any of the HeartBot conversations in the HeartBot dataset (phase 2), and so the model would not fit; thus, we could not really differentiate the intervention effect from the word count.

**Table 4.** Ordinal logistic regression models comparing post- versus pre-intervention (Human or HeartBot) on the 4 knowledge questions^a^.

Cohort	Term	Q1: Recognizing signs and symptoms of a heart attack		Q2: Telling the difference between the signs or symptoms of a heart attack and other medical problems		Q3: Calling an ambulance or dialing 911 when experiencing heart attack		Q4: Getting to an emergency room within 60 minutes after onset of symptoms of a heart attack	
		AOR^b^ (95% CI)	*P* value	AOR (95% CI)^c^	*P* value	AOR (95% CI)	*P* value	AOR (95% CI)	*P* value
Human-delivered conversation	Post (vs pre)	15.19 (8.46, 27.25)	<.001^d^	9.44 (5.60, 15.91)	<.001^d^	6.87 (4.09, 11.55)	<.001^d^	8.68 (4.98, 15.15)	<.001^d^
HeartBot conversation	Post (vs pre)	7.18 (3.59, 14.36)	<.001^d^	5.44 (2.76, 10.74)	<.001^d^	5.74 (2.84, 11.60)	<.001^d^	2.86 (1.55, 5.28)	<.001^d^
All	Post × HeartBot	0.38 (0.19, 0.78)	.008^e^	0.40 (0.20, 0.80)	0.01^d^	0.53 (0.25, 1.10)	.09	0.26 (0.12, 0.55)	<.001^d^

a Models are additionally adjusted for White (vs non-White), age, group type, education, user word count, mean text message effectiveness, and humanness of scores (full models in Multimedia Appendix 2 Table S2); Q1, How sure are you that you could recognize the signs and symptoms of a heart attack in yourself? (Select a number from 1: not sure to 4: sure); Q2: How sure are you that you could tell the difference between the signs or symptoms of a heart attack and other medical problems? (Select a number from 1: not sure to 4: sure); Q3: How sure are you that you could call an ambulance or dial 911 if you thought you were having a heart attack? (Select a number from 1: not sure to 4: sure); Q4, How sure are you that you could get to an emergency room within 60 minutes after onset of your symptoms? (Select a number from 1: not sure to 4: sure).

b AOR, adjusted odds ratio.

c 95% CI, 95% confidence interval.

d ***P<.001.

e **P<.01.

### Human-Delivered Conversation Versus HeartBot Conversation

Table 5 presents the comparison of the evaluation of conversation quality between the 2 studies. In the Human dataset (phase 1), participants interacted with the research interventionist and completed conversation sessions over the course of 2 days. The mean (SD) and median number of words used by the participants and their conversing agent overall were 2322.00 (875.65) and 2097.00 words in the Human dataset (phase 1), and 888.04 (76.04) and 852 words in the HeartBot dataset (phase 2). Participants in the Human dataset (phase 1) ranked all conversational qualities, which include message effectiveness, message humanness, conversation naturalness, and coherence, significantly higher than those in the HeartBot dataset (phase 2). About 74.3% (127/171) and 66.3% (61/92) of the participants in the Human and HeartBot datasets in both groups correctly identified when they were conversing with a human or HeartBot, respectively.

**Table 5.** Comparing the evaluation of conversation quality between the Human dataset and the HeartBot dataset.

	Human dataset (n=171)	HeartBot dataset (n=92)	*P* value
User experience, mean (SD)	<.001
Score of Message Effectiveness scale	6.35 (0.85)	5.66 (1.23)	
Conversation quality (subjective measure)	<.001
Score of Message Humanness scale, mean (SD)	5.86 (1.24)	5.19 (1.19)	
Overall, how would you rate the conversations with your texting partner?, n (%)			<.001
Very unnatural/unnatural	9 (5.3)	5 (5.4)
Neutral	19 (11.1)	33 (35.9)
Natural/very natural	143 (83.6)	54 (58.7)
Overall, how would you rate the messages you received?, n (%)			<.001
Very incoherent/incoherent	1 (0.6)	0 (0)
Neutral	4 (2.3)	23 (25.0)
Coherent/very coherent	143 (83.6)	69 (75.0)
Conversation quality (objective measure), mean (SD)/[range]/median	<.001
Number of words used by the participants and research interventionist/HeartBot	2322.55 (875.65)/ [1314.0‐8073.0]/2097.0	888.04 (76.4)/ [778‐1274]/852	
Number of words used by the participants	298.94 (227.90)/ [83.0‐1986.0]/231.0	80.57 (60.19)/ [34-377]/63	
Do you think you texted a human or artificial intelligent chatbot during your conversation?, n (%)			<.001
Human	127 (74.3)	31 (33.7)
Artificial intelligence chatbot	44 (25.7)	61 (66.3)

## Discussion

### Principal Results

We compared the potential efficacy of human-delivered conversations versus HeartBot conversations in increasing participants’ knowledge and awareness of symptoms and the appropriate response to a heart attack in the United States, while controlling for potential confounding factors. Since this study was not an RCT, the efficacy of the HeartBot intervention, compared to the SMS text messaging intervention delivered by a research interventionist, cannot be established. Caution needs to be exercised when interpreting the findings. The findings suggest that interacting with both the research interventionist and HeartBot was associated with increased knowledge and awareness of a heart attack among participants (ie, recognizing signs and symptoms of a heart attack, telling the difference between the signs or symptoms of a heart attack and other medical problems, calling an ambulance or dialing 911 when experiencing heart attack, getting to an emergency room within 60 minutes after onset of symptoms of a heart attack). However, human-delivered conversations appeared to have a stronger association than HeartBot conversations for all except for the question regarding calling an ambulance (*P*=.09). This may be due to the fact that calling emergency services is a well-known emergency response behavior, which may not require adaptive or relational communication to be effectively understood. Yet, this does not suggest that HeartBot was ineffective. Interacting with HeartBot still led to significant improvements in increasing knowledge and awareness of a heart attack. Given its automated nature and lower cost, we view HeartBot as a promising and useful alternative, particularly in contexts where human resources are limited.

Several potential explanations can be considered due to the fundamental structural differences in the content and duration of the conversation sessions between the 2 studies. First, human-delivered conversations involved a more extended engagement process, comprising 2 separate sessions over a week, allowing participants to engage in a more prolonged and reflective learning process. In contrast, the HeartBot conversation was limited to a single session, which may have constrained the depth of discussion. Second, participants in the Human dataset (phase 1) produced significantly more words during the conversation, with a mean (SD) word count of 298.94 (227.90), compared to 80.57 (60.19) in the HeartBot dataset (phase 2). The greater verbosity in the Human dataset (phase 1) may have contributed to deeper discussions and enhanced knowledge reinforcement, potentially explaining the observed increase in efficacy. However, we were not able to statistically account for word count, as models adjusting for the covariate would not converge, likely owing to having very different distributions of word counts with little overlap in the 2 groups (humans a mean [SD] of 2322.00 [875.65] words, HeartBot a mean [SD] of 888.04 [76.04] words). Finally, human-delivered conversations were facilitated by a research interventionist, who is a master-prepared, cardiovascular nurse, allowing for greater flexibility in language use, response adaptation, and addressing participant queries in a more personalized manner. In contrast, HeartBot had the inherent limitation in its conversational algorithm, which appears less personalized and less flexible, following a structured script, limiting its ability to adjust dynamically to participants’ specific concerns.

HeartBot, a fully automated AI chatbot, was significantly associated with increased participants’ knowledge and awareness of symptoms and response to a heart attack and demonstrates significant potential as an innovative AI intervention. AI chatbots offer a scalable, 24/7 accessible, and personalized approach to health education for broader populations. AI chatbots’ adaptive algorithms allow for dynamic personalization, tailoring responses to individual user queries and comprehension levels, which may enhance engagement and knowledge retention beyond one-size-fits-all campaigns. Additionally, chatbot interactions require active engagement as participants read, process, and respond to information, reinforcing learning through interaction rather than passive intake [36]. The anonymized nature of chatbot conversations can also reduce psychological barriers, encouraging users to seek information more openly, especially on sensitive health topics [37]. Finally, HeartBot integrates structured quiz components, encouraging reinforcement of learning through immediate self-assessment and cognitive recall.

While these advantages highlight AI chatbots’ potential, findings from this study suggest room for improvement to further enhance their efficacy. First, increasing the number of interaction sessions—rather than a single 1-time interaction—may allow for more sustained engagement and deeper knowledge retention, aligning more closely with the multi-session format of human-delivered conversations. Second, further iterations could leverage machine learning algorithms to continuously refine conversation models and improve HeartBot’s flexibility in answering participants’ queries, which could make interaction with HeartBot feel more responsive and personalized. Lastly, to fully evaluate HeartBot’s long-term efficacy and potential parity with human-delivered conversations, a rigorously designed RCT would be instrumental. While this study provides promising preliminary insights, causal relationships cannot be established. Future research should prioritize RCTs to confirm these findings and support evidence-based deployment of such interventions.

Interestingly, user experience and conversational quality were perceived to be high across both studies, as participants generally rated the message as effective, humanlike, coherent, and natural. However, these perceptions were significantly higher in the Human dataset (phase 1). This may be due to participants subconsciously detecting cues that felt more human. Although the identity of the conversing partner was not disclosed, a substantial portion of participants misperceived whether they were interacting with a human or an AI chatbot. While the perception of partner identity was not a primary focus of this study, these misattributions nonetheless provide insight into how users process conversational agency. They highlight the inherent ambiguity in conversational agency and may reflect the challenge of replicating human communication subtleties in algorithmic interactions. While HeartBot demonstrated considerable communicative competence, it encountered limitations in fully imitating the nuanced relational aspects of human dialog. Drawing from the Computers Are Social Actors paradigm [38], participants apply social interaction schemas to technological interfaces yet experience these interactions with less emotional depth and relational intimacy. Key communication studies have consistently highlighted the critical role of relational cues in establishing trust and engagement and promoting human-chatbot relationships. For example, research has shown that conversational agents can build positive relationships in health and well-being settings through verbal behaviors like humor [39], social dialog [40], and empathy [41]. Although HeartBot successfully delivered equivalent factual content, it inherently struggled to reproduce the affective dimensions that characterize human-to-human communication. These findings suggest that while AI chatbots provide a promising technological intervention, they must continue to evolve in their ability to simulate the nuanced relational components of effective human health communication.

### Limitations and Suggestions for Future Studies

Several limitations of this study need to be acknowledged. Without a true RCT, the causal inferences regarding the 2 interventions cannot be determined, and the findings provide only exploratory comparative insights due to the following reasons. The 2 datasets were not collected under a single randomized protocol. Participants were not randomly assigned, making the study vulnerable to selection bias and unmeasured confounders. In particular, human-delivered conversations were much longer (~2322 words) than HeartBot (~888 words). Statistical adjustment was not possible due to nonoverlapping distributions. This is a major confounder that prevents clear attribution of effects to delivery mode versus conversation length. In other words, the differences in exposure length make it impossible to disentangle “agent effect” (human vs HeartBot) from “dose effect” (amount of content). The interventions also differed not only in delivery agent but also in structure: (1) the human-delivered arm included 2 sessions, while the chatbot was a single session; (2) some topics were omitted in the HeartBot group; and (3) incentives differed ($40 vs $20). An RCT addressing these limitations is warranted to validate this study’s findings.

Another limitation is related to the study measures and the timing of the measures. The outcome assessment relied on subjective Likert scale responses, which may be influenced by recall or social desirability bias. Furthermore, the outcomes were assessed between 4 and 6 weeks after the intervention. Thus, the study only captures short-term awareness and knowledge gains rather than sustained retention or behavior change. Future studies need to include objective or performance-based measures (eg, quizzes, simulated scenarios) to complement self-reports, longitudinal follow-up (ie, 2-24 mo) to assess retention, and whether increased awareness and knowledge translate into real-world emergency response behaviors. Additionally, the multinomial mixed effects logistic regression model would not converge. This is a known problem with these models due to a combination of small cell counts in specific outcome categories and the high-dimensional nature of the random effects in the model. However, our more parsimonious ordinal mixed effects logistic regression model converged and appeared to fit the data well.

The last limitation is related to the generalizability of the finding. The current recruitment strategy relied on social media (Facebook or Instagram) and self-selected women who were comfortable with technology. This may skew the sample toward digitally literate participants and limit generalizability to more diverse or higher-risk groups. Thus, future studies should include purposive recruitment strategies targeting underrepresented groups (ie, older women, nondigital populations, and those with lower health literacy).

### Conclusions

The study’s findings provide new insights into the fully automated AI HeartBot, compared to the human-driven text message conversation, and suggest that it has potential in improving women’s knowledge and awareness of heart attack symptoms and appropriate response behaviors. Nevertheless, the current evidence remains preliminary. To rigorously establish the efficacy of the HeartBot intervention, future research should employ RCT designs with the capacity to reach broad and diverse populations.

## Supplementary material

10.2196/73184 Multimedia Appendix 1 Flow diagrams: screening, enrollment, and follow-up of the study participants.

10.2196/73184 Multimedia Appendix 2 Results of full ordinal logistic regression models for each study phase. This appendix includes Table S1 (Full ordinal logistic regression models for Human Text Conversation), Table S2 (Full ordinal logistic regression models for HeartBot Conversation), and Table S3 (Full ordinal logistic regression models for all data).

## Abbreviations

AI: artificial intelligence
AOR: adjusted odds ratio
IRB: Institutional Review Board
RCT: randomized controlled trial
