# Supplementary material for: Human-Delivered Conversation Versus AI Chatbot Conversation in Increasing Heart Attack Knowledge in Women in the United States: Quasi-Experimental Studies
Source: J Med Internet Res. 2025 Oct 17;27:e73184. doi: 10.2196/73184 (PMC12538107; doi:10.2196/73184)

## **Multimedia Appendix 1**.

Figure S1. Flow diagrams: screening, enrollment, and follow-up of the study participants.


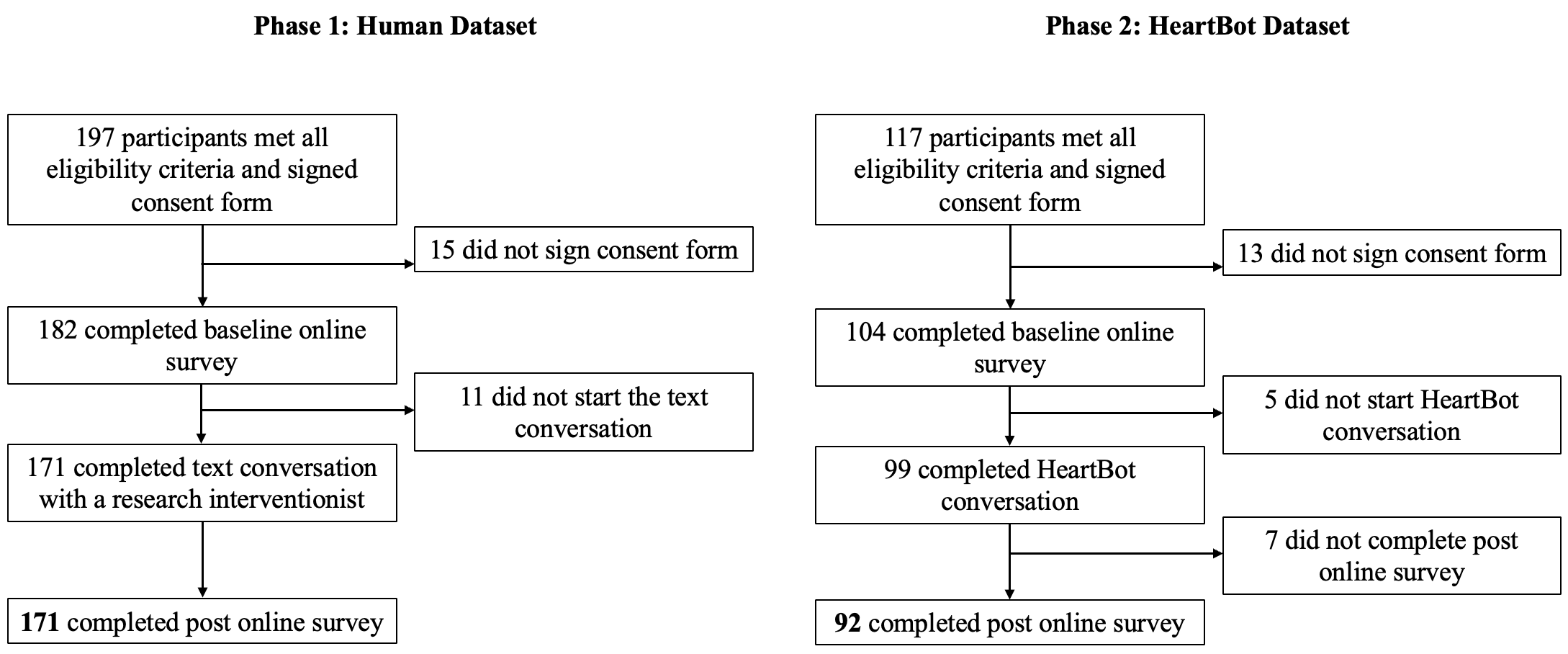

Supplement: Multimedia Appendix 1 [file jmir-v27-e73184-s001.docx]
