# Supplementary material for: Human-Delivered Conversation Versus AI Chatbot Conversation in Increasing Heart Attack Knowledge in Women in the United States: Quasi-Experimental Studies
Source: J Med Internet Res. 2025 Oct 17;27:e73184. doi: 10.2196/73184 (PMC12538107; doi:10.2196/73184)
Supplement: Multimedia Appendix 2 [file jmir-v27-e73184-s002.docx]

## **Multimedia Appendix 3.**

| Table S1. Full ordinal logistic regression models for Human Text Conversation | | | | | | | | |
| --- | --- | --- | --- | --- | --- | --- | --- | --- |
| **Variable** | **Q1: Recognizing signs and symptoms of a heart attack** | | **Q2: Telling the difference between the signs or symptoms of a heart attack and other medical problems** | | **Q3: Calling an ambulance or dialing 911 when experiencing heart attack** | | **Q4: Getting to an emergency room within 60 minutes after onset of symptoms of a heart attack** | |
|  | AOR (95% CI) | *P* value | AOR (95% CI) | *P* value | AOR (95% CI) | *P* value | AOR (95% CI) | *P* value |
| **P: Post (vs. pre) knowledge** | 15.19 (8.46, 27.25) | 7.5e-20*** | 9.44 (5.60, 15.91) | 3.4e-17*** | 6.87 (4.09, 11.55) | 3.5e-13*** | 8.68 (4.98, 15.15) | 2.7e-14*** |
| **Age (years)** | 0.99 (0.97, 1.01) | 0.44 | 0.99 (0.97, 1.01) | 0.32 | 1.01 (0.99, 1.03) | 0.32 | 1.02 (1.00, 1.05) | 0.059 |
| **Non-white (vs. white)** | 0.90 (0.52, 1.58) | 0.72 | 1.30 (0.71, 2.40) | 0.4 | 1.36 (0.77, 2.41) | 0.3 | 0.50 (0.26, 0.98)* | 0.045 |
| **Education (completed collage/graduate school vs. not)** | 0.64 (0.37, 1.09) | 0.098 | 0.58 (0.32, 1.03) | 0.064 | 0.63 (0.36, 1.11) | 0.11 | 0.72 (0.39, 1.32) | 0.29 |
| **Message effectiveness^a^** | 0.84 (0.57, 1.25) | 0.4 | 0.84 (0.55, 1.30) | 0.44 | 0.95 (0.63, 1.43) | 0.81 | 0.98 (0.63, 1.54) | 0.94 |
| **Message humanness^a^** | 1.36 (1.03, 1.79)* | 0.029 | 1.33 (0.99, 1.80) | 0.061 | 1.19 (0.90, 1.58) | 0.23 | 0.98 (0.72, 1.34) | 0.9 |
| **Chatbot use history** | 0.99 (0.62, 1.60) | 0.98 | 0.95 (0.57, 1.60) | 0.85 | 0.80 (0.49, 1.33) | 0.4 | 0.96 (0.55, 1.67) | 0.89 |

**Footnote.** Q1, How sure are you that you could recognize the signs and symptoms of a heart attack in yourself? (Select a number from 1: not sure to 4: sure); Q2: How sure are you that you could tell the difference between the signs or symptoms of a heart attack and other medical problems? (Select a number from 1: not sure to 4: sure); Q3: How sure are you that you could call an ambulance or dial 911 if you thought you were having a heart attack? (Select a number from 1: not sure to 4: sure); Q4, How sure are you that you could get to an emergency room within 60 minutes after onset of your symptoms? (Select a number from 1: not sure to 4: sure); *, P<0.05; **, P<0.01; ***, P<0.001. ^a^Message effectiveness and message humanness each consist of 5 items, and each item scores are based on a 7-point Likert scale. The scores of the 5 items for message effectiveness and message impression were summed and averaged to create a mean composite score (Cronbach α=0.94 and 0.91, respectively); Abbreviation: AOR, adjusted odds ratio; 95% CI, 95% confidence interval.

| Table S2. Full ordinal logistic regression models for HeartBot Conversation | | | | | | | | |
| --- | --- | --- | --- | --- | --- | --- | --- | --- |
| **Variable** | **Q1: Recognizing signs and symptoms of a heart attack** | | **Q2: Telling the difference between the signs or symptoms of a heart attack and other medical problems** | | **Q3: Calling an ambulance or dialing 911 when experiencing heart attack** | | **Q4: Getting to an emergency room within 60 minutes after onset of symptoms of a heart attack** | |
|  | AOR (95% CI) | *P* value | AOR (95% CI) | *P* value | AOR (95% CI) | *P* value | AOR (95% CI) | *P* value |
| **P: Post (vs. pre) knowledge** | 7.18 (3.59, 14.36) | 2.5e-08*** | 5.44 (2.76, 10.74) | 1.1e-06*** | 5.74 (2.84, 11.60) | 1.1e-06*** | 2.86 (1.55, 5.28) | 0.00078*** |
| **Age (years)** | 0.97 (0.93, 1.00) | 0.055 | 0.97 (0.94, 1.01) | 0.16 | 0.97 (0.94, 1.01) | 0.16 | 1.04 (1.00, 1.07) | 0.044* |
| **Education (completed collage/graduate school vs. not)** | 0.35 (0.14, 0.87) | 0.025* | 0.19 (0.07, 0.51) | 0.00099*** | 0.56 (0.21, 1.48) | 0.24 | 0.37 (0.15, 0.93) | 0.035* |
| **Message effectiveness^a^** | 1.60 (1.03, 2.48) | 0.037* | 1.62 (1.02, 2.59) | 0.042* | 1.81 (1.09, 3.00) | 0.022* | 1.38 (0.90, 2.11) | 0.14 |
| **Message humanness^a^** | 1.20 (0.77, 1.87) | 0.42 | 1.06 (0.67, 1.68) | 0.81 | 0.91 (0.55, 1.53) | 0.73 | 1.24 (0.79, 1.94) | 0.00078 |
| **Chatbot use history** | 1.32 (0.60, 2.87) | 0.49 | 1.71 (0.75, 3.87) | 0.2 | 0.89 (0.38, 2.09) | 0.8 | 0.86 (0.40, 1.86) | 0.044 |

**Footnote.** Q1, How sure are you that you could recognize the signs and symptoms of a heart attack in yourself? (Select a number from 1: not sure to 4: sure); Q2: How sure are you that you could tell the difference between the signs or symptoms of a heart attack and other medical problems? (Select a number from 1: not sure to 4: sure); Q3: How sure are you that you could call an ambulance or dial 911 if you thought you were having a heart attack? (Select a number from 1: not sure to 4: sure); Q4, How sure are you that you could get to an emergency room within 60 minutes after onset of your symptoms? (Select a number from 1: not sure to 4: sure); *, P<0.05; **, P<0.01; ***, P<0.001. ^a^Message effectiveness and message humanness each consist of 5 items, and each item scores are based on a 7-point Likert scale. The scores of the 5 items for message effectiveness and message impression were summed and averaged to create a mean composite score (Cronbach α=0.94 and 0.91, respectively); Abbreviation: AOR, adjusted odds ratio; 95% CI, 95% confidence interval.

| Table S3. Full ordinal logistic regression models for all data | | | | | | | | |
| --- | --- | --- | --- | --- | --- | --- | --- | --- |
| **Variable** | **Q1: Recognizing signs and symptoms of a heart attack** | | **Q2: Telling the difference between the signs or symptoms of a heart attack and other medical problems** | | **Q3: Calling an ambulance or dialing 911 when experiencing heart attack** | | **Q4: Getting to an emergency room within 60 minutes after onset of symptoms of a heart attack** | |
|  | AOR (95% CI) | *P* value | AOR (95% CI) | *P* value | AOR (95% CI) | *P* value | AOR (95% CI) | *P* value |
| **P: Post (vs. pre) knowledge** | 16.29 (9.40, 28.20) | 2.3e-23*** | 11.08 (6.68, 18.37)*** | 1.1e-20*** | 8.25 (4.91, 13.86)*** | 1.5e-15*** | 9.79 (5.72, 16.75) | 8.2e-17*** |
| **H: Heartbot vs. human** | 0.97 (0.53, 1.79) | 0.92 | 1.25 (0.66, 2.37) | 0.5 | 1.38 (0.75, 2.53) | 0.3 | 1.45 (0.77, 2.71) | 0.25 |
| **P x H interaction** | 0.38 (0.19, 0.78) | 0.0084** | 0.40 (0.20, 0.80) | 0.01* | 0.53 (0.25, 1.10) | 0.089 | 0.26 (0.12, 0.55) | 0.00044*** |
| **Age (years)** | 0.98 (0.97, 1.00) | 0.084 | 0.99 (0.97, 1.01) | 0.17 | 1.00 (0.98, 1.02) | 0.91 | 1.03 (1.01, 1.05) | 0.0045** |
| **Non-white (vs. white)** | 0.85 (0.47, 1.55) | 0.6 | 1.17 (0.62, 2.20) | 0.62 | 1.47 (0.80, 2.71) | 0.22 | 0.41 (0.21, 0.79) | 0.0082** |
| **Education (completed collage/graduate school vs. not)** | 0.55 (0.34, 0.89) | 0.015* | 0.42 (0.26, 0.70) | 0.00075*** | 0.64 (0.39, 1.05) | 0.08 | 0.58 (0.35, 0.96) | 0.034* |
| **Message effectiveness^a^** | 1.21 (0.91, 1.60) | 0.19 | 1.17 (0.86, 1.58) | 0.31 | 1.30 (0.96, 1.75) | 0.085 | 1.24 (0.92, 1.68) | 0.16 |
| **Message humanness^a^** | 1.23 (0.97, 1.55) | 0.087 | 1.17 (0.91, 1.50) | 0.22 | 1.06 (0.83, 1.36) | 0.64 | 0.99 (0.77, 1.28) | 0.97 |
| **Chatbot use history** | 1.02 (0.67, 1.54) | 0.94 | 1.09 (0.70, 1.69) | 0.71 | 0.77 (0.50, 1.19) | 0.24 | 0.89 (0.57, 1.39) | 0.61 |

**Footnote.** Q1, How sure are you that you could recognize the signs and symptoms of a heart attack in yourself? (Select a number from 1: not sure to 4: sure); Q2: How sure are you that you could tell the difference between the signs or symptoms of a heart attack and other medical problems? (Select a number from 1: not sure to 4: sure); Q3: How sure are you that you could call an ambulance or dial 911 if you thought you were having a heart attack? (Select a number from 1: not sure to 4: sure); Q4, How sure are you that you could get to an emergency room within 60 minutes after onset of your symptoms? (Select a number from 1: not sure to 4: sure); *, P<0.05; **, P<0.01; ***, P<0.001. ^a^Message effectiveness and message humanness each consist of 5 items, and each item scores are based on a 7-point Likert scale. The scores of the 5 items for message effectiveness and message impression were summed and averaged to create a mean composite score (Cronbach α=0.94 and 0.91, respectively); Abbreviation: AOR, adjusted odds ratio; 95% CI, 95% confidence interval.
